# The Temporal Uses of Moral Things: Manifesting, Anchoring and Conserving Caring Relations within the Sensorium

**DOI:** 10.1177/0038038520959263

**Published:** 2020-12-04

**Authors:** Andrew Balmer, Robert Meckin, Owen Abbott

**Affiliations:** University of Manchester, UK; University of Manchester, UK; University of Manchester, UK

**Keywords:** care, everyday life, material culture, morality, practices, sensory, smell, taste, temporality, time

## Abstract

In this article we argue that menthol-containing products, like chewing gums, vapour rubs and mouthwashes, are used as *moral things* within everyday practices. They take on moral functions because of how their material qualities contribute to sensory experiences. Specifically, we focus on scenarios in which menthol products become associated with the moral work of care and highlight the temporal dimension of what people do with moral things. We review the literature on morality as a practical, everyday accomplishment and stress the embodied nature of caring practices to outline how care is bound up with sensory experience. We draw on rich qualitative data generated through creative methods, including film, photography and sketching, as part of object-elicitation interviews, focus groups, home tours and ‘pop-up stalls’. We develop three concepts regarding the function of moral things: manifesting, anchoring and conserving moral relations to describe how time, morality and the sensory are entwined.

## Introduction

In this article we explore how people use products that contain menthol, the molecule in mint which produces a distinctive taste, smell and sensation in the throat, mouth and on the lips, on the skin and sometimes even in the eyes. Alongside lemon and vanilla, it is among the most popular chemical flavours and fragrances in mundane consumer goods in the West. It is an ingredient in many products, including toothpastes, mouthwashes, muscle rubs, skin and lip balms, shower gels, shampoos, cold remedies, medicines, confectionery and chewing gums. Indeed, when participants in our study viewed the range of goods in which menthol was an ingredient, they were often surprised at their number and at the diversity of menthol’s uses (Meckin and Balmer, 2019). This surprise points to both the ubiquity and invisibility of menthol – it is so commonplace it is mostly experienced as an inconspicuous part of our everyday lives, from brushing our teeth in the routine of waking up the body in the morning, to putting a dab of scented oil on a child’s pillow to sooth them in an evening parenting routine.

A peculiarity of menthol is that the human body can distinguish the cooling sensation and minty taste even when menthol is mixed with other flavours, salts and stabilising chemicals in these products. A similar kind of cool tingle is experienced, whether on the scalp from washing hair with mentholated shampoo, or on the back of the throat from sucking a potent lozenge. These products span, on a regulatory axis, from not quite foods (like chewing gum) to not quite medicines (like mentholated cough mixtures). The variety of products in which menthol figures means that the minty, cooling, tingling sensation is implicated in an array of diverse practices and relations, each involving different combinations of skills, ethics and know-how for their successful performance and management (Meckin and Balmer, 2018).

To explore how people use menthol we draw on rich qualitative data generated through creative, visual methods, including video, photography and sketching, made while engaging participants in object-elicitation activities. We conducted elicitation interviews with people using menthol objects that we brought to them, or which they brought from their homes, offices and vehicles; we followed people around as they toured us through their homes showing us where menthol products were situated; and we used a mass object-elicitation technique that we called ‘pop-up stalls’ to engage hundreds more people in producing data about how menthol figures in our routine practices, biographies and everyday relationships (Meckin and Balmer, 2017).

In exploring the uses of this mundane molecule, we seek to investigate sociological questions concerning everyday moral practices. Specifically, we focus here on some of the moral practices of care, in which we discovered menthol commonly played a role. Participants describing their use of chewing gum, for example, would often appeal to the social norm of having fresh breath as something that we could all understand, inasmuch as it was each individual’s responsibility to keep their breath fresh. As such, we conceptualise menthol products as ‘moral things’, objects which help achieve moral work by virtue of their sensory, material qualities. To focus our analysis, we constrain our considerations of the moral work people do to the specific case of care as a routine moral practice.

As we analysed our data, what we also began to realise was that such articulations about moral uses of menthol depended upon temporal organisation. For example, they spoke to a kind of sensory upkeep in which the timing of menthol’s uses seemed to be tied to the rhythms of moral life. A person might chew gum in the bathroom of a restaurant after eating a meal with a romantic partner, in anticipation of sharing a kiss. Or an employee might chew gum in their car when driving from one meeting to another, to refresh their breath after drinking a coffee. In these simple examples, we saw a broader question: how is the moral and temporal order linked in the use of such moral things?

We begin our development of this question regarding the uses of moral things by reviewing some of the literature on everyday moral order and embodiment, selectively focusing on how care is conceptualised within this literature. We then discuss scholarship on the senses, with regard to a concern for articulating how sensory phenomena are bound up with moral life. In the empirical section, we discuss three concepts that we generated from the data analysis, focusing on how each links time and morality: *manifesting* relates to moral work that occurs during moments of interactive performances; *anchoring* is a residual moral effect that hinges on how sensory experience can linger on after such performances have finished; and finally, *conserving* relates to the way participants pass on and reproduce temporal-moral practices by preserving sensory meanings for the next generation.

## Embodied Moral Practices of Care in the Sensory World

Over the past two decades the sociological analysis of morality has received renewed attention, reconceiving morality not as something abstract but as something that is both variably and ordinarily enacted within everyday social practices. Morality, in this approach, is understood to have palpable significance to the narrative accounts people give of themselves, their actions and their relations with others (Abbott, 2020; Finch, 1989; Hitlin and Vaisey, 2013; May, 2008). One notable development resulting from this reconceptualisation has been the expansion of moral practices to include many everyday phenomena, such as routine forms of care for oneself and others; concern for relationships; situated responsiveness to interpersonal interactions; and the mundane enactment of what is subjectively perceived to be the ‘proper’ thing to do. Gilligan’s (1982) challenge to the orthodoxy of moral philosophy is emblematic of this renewed attention as she explicates precisely why everyday relations of care are important moral concerns at the heart of ethical life rather than issues of peripheral interest. This has been ‘vital in the re-evaluation of “care” as a form of ethical activity and moral thinking’ (Smart and Neale, 1999: 115). In conducting our project and in analysing our data we found that care was a key moral practice in which menthol often played a role. To explore this finding, we take inspiration from this existing body of scholarship, seeing care as an important moral activity in everyday experience. In this section we use this literature to show how morality and sensory phenomena are connected in everyday life but identify a key gap in this work, namely with regard to the temporal dimensions of moral experience.

Care as a routine moral practice must be understood as a fundamentally embodied, practical phenomenon. For example, Dreyfus and Dreyfus (2014) emphasise that everyday ethical comportment in the social world involves a skilful embodiment of concern for those with whom we interact, evidencing a dispositional alignment of our bodily actions with commonplace expectations and values of decency. This has some affinity with approaches to the moral order deriving from Goffman’s (1959) work, in which the performance of an acceptable self in interaction reflects a habituated concern to present oneself as being of a sound character, in line with the ordinary moral expectations of our social world. In arguing that the performance of the self reflects an ordinary concern for moral order, Goffman characterised the body as a repository of social meanings through which moral acceptability and transgression are imparted (Abbott, 2020; Crossley, 1995; Low, 2009).

Such embodiment helps us to see how the sensory dimensions of everyday life are inextricably entangled with the moral order (Low, 2009; Shilling, 1993; Vannini et al., 2012). Literatures in various fields of social research have explored the links between the human senses and morality (Classen et al., 1994; Howes, 2003; Pink, 2015; Synnott, 1993). Although sensations often appear to be ‘naturally given’ they are actively constituted in practices that include aesthetic and moral components (Waskul and Vannini, 2008). Of the sensations, the sense of smell is arguably most intimately connected to moral practices of gender, class, ethnicity and work (Corbin, 1986; Largey and Watson, 1972; Skeggs, 2005). With respect to caring relations in particular, Mann (2018) demonstrates how micro-practices organise sensory experiences via an embodied concern for the interactional order. Thus, actions upon the sensorium become vectors through which morality is enacted. This helps us to conceptualise care as a material and relational moral practice making it tractable with the data we created through use of a sensory methodology (which we outline in the next section). As we began to explore this connection between morality and the senses in our data set, we found that time kept cropping up in one way or another, but we could not find adequate tools in the literature to analyse this set of connections.

Indeed, it is often taken to be self-evident that moral practices occur and unfold in time (see, for example, Dreyfus and Dreyfus, 2014), and sociological research has explored how such practices can be directed towards different temporal orders, for example in the remembering of loved ones or prudently planning for a child’s future (Sayer, 2005). Likewise, sociological work has explored how uses of time are morally ordered (Slater, 2009), and how routine practices are often organised and adjusted in order to ‘make time’ for caring relations to be fulfilled and extended (Shove, 2009; Southerton, 2003). Ethical consumption literatures have highlighted that morally sound practice is tied to the temporality of products themselves, for example by buying at the ‘right time’, using up ‘in time’ before produce goes bad and freezing and pickling to ‘extend time’ of usability (Mattila et al., 2019). Here we begin to see how the material affects the moral within the temporal dimension, which looks promising for our concern with menthol. Elsewhere, the temporal-moral significance of materiality is explored through research into heirlooms and gifts that mark significant life events, demonstrating how the temporal orientations of such materials allow them to be embedded with moral significance (Jalas, 2009; Shove, 2009). In this way, objects can be imbued with moral significance which might carry across time, and this gets closer to the sorts of moral scenarios we discovered in our data. However, while arguments like these have been made about moral practices being temporally contextualised, with indications that the material might sometimes play a role in this connection, few attempts have been made to *conceptualise* the temporal qualities of material objects in a fashion that might explain how it is that some kinds of moral work are achieved temporally more generally.

One of our key findings in our data analysis is that time, morality and the sensory are importantly woven together in ways that depend upon the specific qualities of the sensory things entangled in these situations. Our article seeks to develop these literatures by tracking some of the moral work achieved by use of a single chemical in different temporal schemes. We thus argue that menthol products are examples of *moral things*, a term which we use to diagnose those material objects that provide a sensory medium through which moral expectations of oneself and others are understood and performed. Specifically, we explore how moral things work in three temporal modes, which we term manifesting, anchoring and conserving.

## Methods

The data we draw upon in developing our argument were collected as part of a three-year project exploring the current uses of menthol-containing products, and whether and how changes in the manufacture of menthol (moving to new kinds of biosynthetic production processes) might lead to changes in people’s understandings and practices. While we had some common-sense ideas about menthol products, the overall research project was exploratory in nature and we did not know the diversity of products and practices at the outset and how they would link to time.

In our methodology we placed an emphasis on understanding differences between participants’ uses and understandings of menthol, while also looking for similarities across the data. We used a range of methods to help us to collect data from a large number of participants, some with only a momentary engagement and others with hours-long discussions and interactions. Practically, we asked people if we could look at their menthol products, or made use of two very large shopping bags of menthol-containing products that we carried around to various sites of engagement and that we used as ethnographic material ‘probes’ (Woodward, 2016). We trialled sensory elicitation in different contexts and with different group sizes:

● We conducted seven one-on-one object-elicitation interviews, where participants looked through and used some of the objects in our shopping bags, as well as bringing along some of their own products. These helped us to understand the ways in which menthol figured within participants’ stories and biographies, for they often focused on memories of using menthol as well as on current uses.
● We did nine home tours (where there were between one and four participants at each location) during which people guided us around their home, showing us where menthol products ‘lived’ and how they used them. This helped us to better understand how menthol figures within everyday interactions and routines of the home and work.
● We organised two focus groups, one with a predominantly academic and middle-class group of attendees and one with a more diverse set of people hosted at a climbing centre. These explored the similarities and differences of menthol practices and facilitated exploration and contestation of different experiences.
● We also experimented with a novel method, which we termed ‘pop-up stalls’, of which we conducted five. These involved us setting up a table of products in a public place: a museum, a university department open day with college-age students, a large shopping centre, a community garden centre and a university ‘taster’ day for school-age students. We attracted the attention of passers-by and, following our initial exchange of some information about what we were doing, asked them to tell us about the products they used and how they used them. Sometimes we built up a rapport which facilitated participants trying some of the products in one way or another (for example eating a cough sweet) and sometimes people picked up and used products without prompting (though we think this almost always happened when they had witnessed other participants using the objects). We made our own notes, and asked all participants to fill out postcards about what they had told us. We collected over 450 postcards which were later transcribed.

The focus groups and pop-up stalls enabled us to understand menthol within interactional situations but also to get at some of the diversity of uses and meanings, since we were able to talk to hundreds of people. We combined audio recording of talk during the use of these elicitation methods with the creation of photographs, video recordings and with field sketching. Although we did some sketching ourselves, most sketches were done by a professional artist, Lynne Chapman, who worked with us on the project for a number of the pop-up stalls and the focus groups (Heath et al., 2018). We thus had visual data of people touching, tasting and smelling menthol-containing products, as they narrated their experiences, feelings, sensations and memories. The audio recordings were professionally transcribed. The field notes, diaries, photographs, videos and transcripts were loaded into NVivo and thematically coded. We discussed the codes and possible connections with our wider research group.

Most of the data were collected in the first half of 2017. Participants who we audio recorded ranged from five years old (supervised by parents) to 60 years old. At the pop-up stalls, however, we believe that some people were older than 65, though they did not give their age. The majority of the interview and home tour participants were white, but – without having captured data on demographics – we believe the pop-up stalls were more reflective of the ethnic diversity in the Greater Manchester region. A key consequence is that our cases were predominantly Western examples of menthol’s use and there may be significant other uses we were unable to capture. In addition, there are likely to be differences in how care is conducted in sub-cultural groups within the UK and by families and friends from different ethnic and religious traditions. Nonetheless, we expect to find that the higher order usage of these products to achieve temporal aspects of moral work would still be present in a diverse range of cultures and scenarios. Of course, this is only speculation based on a few cross-cultural encounters we had during our data gathering and in the period since.

The study received ethical approval from the University of Manchester Research Ethics Committee. Researching from a sensory perspective comes with an ethical framework that, while broadly in line with qualitative research ethics, has peculiarities of its own for which reflexivity becomes especially important (Pink, 2015). For instance, while sensory experience can be evocative and ‘transport’ participants to the past this evocation is often not benign or neutral and can be loaded with potent experiences and meanings. So, reflexivity and awareness of the potential for producing vulnerabilities of painful or otherwise upsetting recollections was needed by the researchers, as was an approach that privileged the participants’ explanatory responses. Second, cultivating the sensory within research methods might help people to articulate or otherwise express important elements of their experience which would otherwise go unnoticed, opening up opportunities to better represent their lives within sociological scholarship, which in itself might be ethically salient.

The simultaneous use of digital recording technologies also created vulnerabilities in our project, especially when generating images of people’s faces. We addressed this by having people give consent to participation in the project and separate consent to use of their images through the use of media release forms. The carers of young people were repeatedly contacted to check they still gave permission for the use of children’s images when we began the publication process. Furthermore, we have chosen to pixelate the faces of identifiable children and people in the background from whom we were unable to obtain consent.

As part of the writing process we refined, adapted and generated new connections in the data and checked the ideas that we developed with each other and against the transcripts, notes and pictures. As a rough account, we began to find morality in the data collection process and started to read the literature on morality and the senses. Early in the data analysis we found that there was also a temporal element to people’s accounts – most immediately obvious in stories about family biography, for example. This developed into a thematic coding of the data under the terms of ‘manifesting and demonstrating’ moral comportments, which coalesced into ‘manifesting’. These terms were quite ‘close’ to user accounts, for example in how someone might say ‘demonstrating I care’. As we coded and explored the literature, we found we did not have an existing set of concepts to deal with the things people were reporting and which we observed with regard to morality, sensory objects and time. As such, we began to treat manifesting as a conceptual articulation that encompassed demonstrating and we developed the separate notion of ‘anchoring’ through our discussions of the data. The identification of these two concepts led us to focus on the temporal dimensions of moral things in a third reading of the data and, with this in mind, we were able to discover a further category, conserving, frequently occurring in the data.

In our subsequent sections we have selected a range of photographs, video stills, excerpts from transcripts and reflections that convey the best possible sense of these concepts. The concepts are at a second order of abstraction that, even though there is a large amount of variation in the first-order analysis of *how* people responded to menthol, what they were achieving at the practice level had a certain amount of consistency. As mid-range concepts, we argue in the conclusion, we anticipate these being of use to other researchers interested in material culture, sensory life, moral practices and temporality.

## Manifesting Care within Gendered Caring Relations

Smell is regularly associated with morality. As Synnott (1993: 193) argues, ‘expenditure on colognes, perfumes, after-shave and other fragrances is not only an investment in the presentation of self, but it is also a major component in the moral construction of the self’. Menthol does not often figure in everyday life as a pleasing smell for the presentation of a competent moral self, except where minty smell is used expressly to cover bad breath, for instance. Nonetheless, it is entangled with self-care and we explore two instances in which self-care was manifested by use of menthol products and show how this was bound up with the broader moral order regarding gender. Our aim is not to contribute significantly to understandings of gender but, instead, to show that manifestations of sensory care have contradictions and differences at one level, but similarities in how they do the moral temporally. This helps us to exemplify our first temporal concept, manifesting, which focuses on morality in the instance of performance.

**Figure 1.**
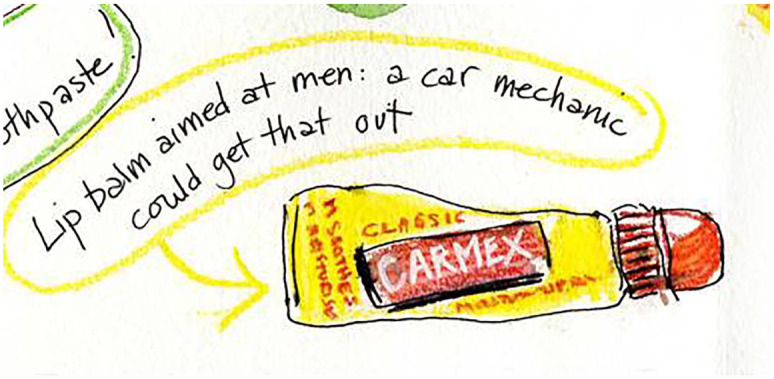
Carmex branded in yellow and red, unlike most menthol products.

Menthol products in the main are not branded towards men. They emphasise ‘soothing’, ‘cooling’ and ‘relief’ and are predominantly aimed at women, families or are ostensibly gender neutral. Recently, this emphasis can be seen by counter-example, in the appearance of a few products specifically targeted at men, including a brand of menthol-containing shower gel which was advertised as producing a ‘tingly balls’ sensation (from menthol’s interaction with sensitive skin on the testicles). The branding of menthol is thus sometimes strategic in its presentation of sensory qualities, as one focus group participant commented when picking up a stick of Carmex, ‘A car mechanic could get that out.’ (See Figure 1 of a sketch from an object-elicitation focus group.) The choice of the car mechanic as characterising a certain type of masculinity indicates, by contrast, the usually feminine or neutral nature of menthol products used in self-care routines.

Although masculinity is performed differently by different men in different situations, it is often associated with self-reliance, strength, power, toughness and invulnerability (Connell, 1995; Courtenay, 2000). As moral life is part of the weave of everyday experience, it too is entangled with performances of gender. Care is an excellent example of one such deeply gendered practice. We explore manifesting as a temporal concept for understanding morality as something being explicitly performed in the moment for participants to a situation.

**Figure 2.**
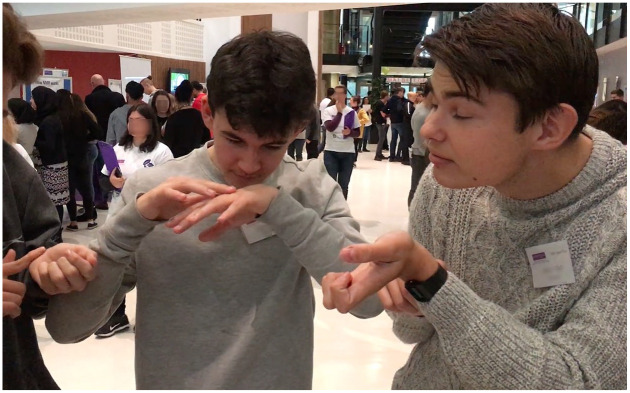
Image still from a video of teenage boys at a pop-up stall mimic smelling perfumes using vapour rub.

Masculinity figured in a memory of menthol for one of the authors, Robert, who did not grow up in a home that used menthol products, so the practices described by participants in the course of data collection were largely alien to him. Before engaging in the project, his most memorable experience was at school, with Tiger Balm, a menthol-containing ointment often used to ease headaches or muscle tiredness. During break time, at secondary school in the mid-1990s, Robert observed a group of male students recovering after smearing Tiger Balm under their eyes. Stood in a circle, they all had streaming red eyes and blotchy cheeks. They pointed to each other’s faces, and commented on their appearances and tears. They were all laughing. In the course of the research, during the pop-up stalls, we (the researchers) would often talk about our own memories of using menthol. Robert told this story to a group of teenage boys at a pop-up stall, and the boys immediately took up the vapour rub and began smearing it on themselves and each other. First, they put it on their wrists and necks mimicking perfume, and then did comical faces pretending to appreciate the smells in a comedic performance of what we took to be their received images of women’s perfume practices (see video still in Figure 2).

**Figure 3.**
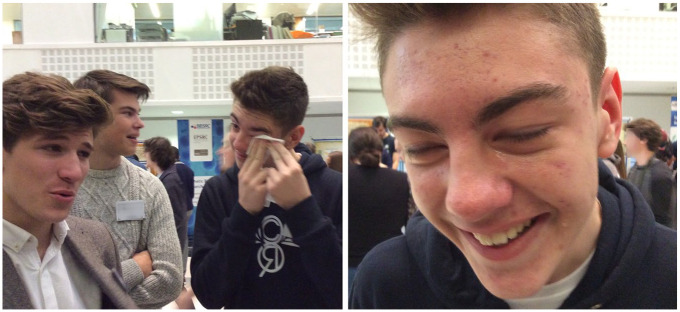
Teenage boys recover after playing a game of menthol one-upmanship, and a close-up of tears streaming down a young man’s face.

Shortly after, to contrast with these gentle approaches to smell, they began smearing more than ample amounts of menthol vapour rub under their eyes in a game of one-upmanship, as each young man attempted to tolerate more of the menthol than the others had previously. The result was much laughing through a lot of streaming tears (see Figure 3).

Menthol’s effects on different bodily surfaces play a role in different kinds of embodied experience, from the cooling sensation on the back of the throat which we usually take to feel ‘clean’ and ‘soothing’, to – in this instance – the burning sensation on the sensitive tissue under the eyes. Managing menthol successfully on the body thus requires some care in order to avoid hurting oneself. For the young men engaged in our pop-up stall, the material, sensory dimensions of vapour rub provided a means to competitive playfulness around their toughness. Lyman (1998) argues that such games allow men to negotiate their affection, tension and aggression towards each other, for example, as can be seen in the context of American frat culture. For our participants, menthol was used to secure the moral order around masculine performances of care, but via the inversion, and thus manifestation, of the ordinary imperative of avoiding self-harm. Manifesting for others, in the form of tears and blotched cheeks, their temporary refusal of their moral obligation to themselves, the young men negotiated the boundaries and tolerances of their bodies’ toughness, through play. Harming themselves with menthol sat in clear distinction to their previous performance of more ‘feminine’ ways of appreciating the products. In doing so, they used menthol to display for each other, and for the researcher who had told the story in the first place, their conformation to idealised notions of masculinity.

**Figure 4.**
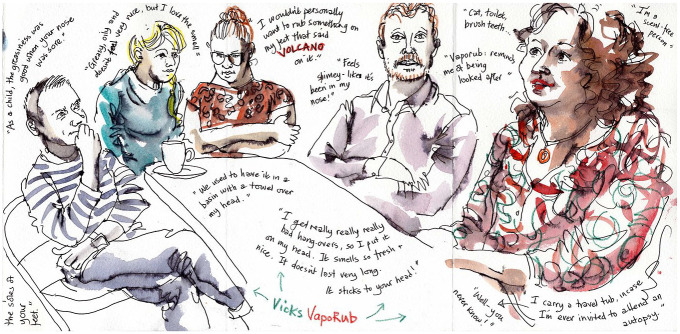
Participants in a focus group discuss menthol products.

In contrast, women in our data were generally far more comfortable linking the use of menthol products to the emotional, sensory and pleasurable dimensions of self-care and we recorded no instances of women using menthol products to demonstrate toughness or a lack of care for their bodies. For example, in a focus group at a climbing centre (see Figure 4), two women, Sue and Kelly, outlined the ways in which they use vapour rubs when they’re suffering from headaches:

Sue: I use it for when I need to put it on my head [with a headache] because it’s really cold and it’s just like that five-minute relief. I think it smells really fresh and nice unlike me at that time! [indicating a hangover] Yes, so I think it just, it’s the smell that makes me feel a bit better – like a cold compress . . . So only for a minute, but I think that is why the texture is good because it just sticks to your head. I have tried alcohol hand gel before, but it doesn’t stick as good.

Interviewer: You have had enough of alcohol! [laughing]

Sue: Probably because I was objecting to the alcohol in it! [agreeing and laughing]

Kelly: I have not used it for ages, but if I had a really bad cold I would definitely use it, yes. I think sometimes you don’t want to be chucking back tablets, that is when I can use it. Just, I don’t know, really to cuddle me through it a little bit. Do you know what I mean?

Sue’s description invokes smell as being relieving. As she holds, smells and touches the vapour rub, the tingle feeling is fresh and nice on her head, which is about feeling better, if only for a minute. Kelly contrasts using menthol products to ‘chucking back’ tablets, in a phrasing which indicates that using medication in that way would be a kind of carelessness. She follows this up with a description of using menthol vapour rub as a ‘cuddle’, clearly invoking a metaphor of being cared for. In this talk, care is not so much about fixing the problem in an instrumental way, since the effects might last only for a minute, but instead is about care as a kind of compassion, gentleness and kindness, in this case, to oneself. The moral status of self-care is demonstrated in Sue and Kelly’s exchange through use of language (contrast, metaphor) and affect (laughter). In this regard, the women demonstrate their own gendered approach to care, highlighting the appreciation of pleasure as a part of care, but also demonstrating their ability to use menthol products in a way which displays the traditional moral role of women as carers. In this way, the participants in the focus group describe what care *should* feel like, in our terms, how it should be manifested. In these participants’ lives, menthol’s coolness on the skin takes on a role in moral manifestation, if only to oneself, of care for oneself, if only for a moment.

In these contrasting examples of using the same menthol product in two differently gendered ways, we see how care becomes manifested by virtue of the sensory dimensions of the vapour rub. The cooling or burning sensation, bound to embodied, gendered norms, is able to effect a moral display of care for oneself and others. Moral things are used to manifest moral order: the sensory and material are used within situations of everyday life to make *sensible* the moral order in apposite ways. What is otherwise ephemeral, invisible or inexplicable, becomes available to touch, taste and smell (boys demonstrating toughness through teary eyes; women demonstrating gentleness through sensory cuddles); a shared understanding of and commitment to moral relations (in this context, of care) is manifested, helping to reaffirm moral organisation within the situation.

## Anchoring and Conserving Care within Family Relations

Gilligan’s (1982) work foregrounded consideration of everyday relationships as a primary locus of moral concern. Research into family and personal life has investigated how practices of care are vital to how personal relationships are negotiated and sustained (Holdsworth and Morgan, 2007; May, 2008). As such, we might expect to find smell, taste and touch playing a role within moral work at this level of everyday relational experience. A focus on the senses also draws attention to how the human body is reciprocally connected into its environment, such as at home. According to Vannini et al. (2012), people act on their environment in attempts to align their sensory experiences with their expectations. They offer an example of a parent soothing a crying child in the night:

It is also an act generating another act: our rising from the bed to attend to the child – either through a sound act like ‘shhh!’ or different responses like feeding – to extinguish an unwanted sound and return the world of the night to the desired sonic order . . . By acting on the sensory world, we negotiate and manipulate the somatic order of a particular situation, and bring it into alignment with the ideal . . . An aligning sensory act is a joint act that enables participants to order their sensescapes and restore sensory order. (Vannini et al., 2012: 136–137)

In the above quotation, the authors make sound, and the restoration of (sonic) order, an important aspect of parental care during the night-time hours. Similarly, parents in our study used menthol to add sensory elements to evening routines. We found that within caring relations, smell and the sensations of tingling, warmth and coolness associated with smelly menthol products, were used to materialise care for others. Many menthol-containing products can be deployed in such practices. These formulations tend to include camphor and eucalyptus oil, too, creating a sensory experience for people of skin tingling, warmth, opening up airways and sensations which most people in our study took to be comforting. Vapour rubs and oily inhalants were especially significant in this regard, and in this section we concentrate predominantly on the ways that parents care for their children and how the material, sensory qualities of these moral things were bound up with the temporally immediate, the proximal and the distant experience of caring relations.

**Figure 5.**
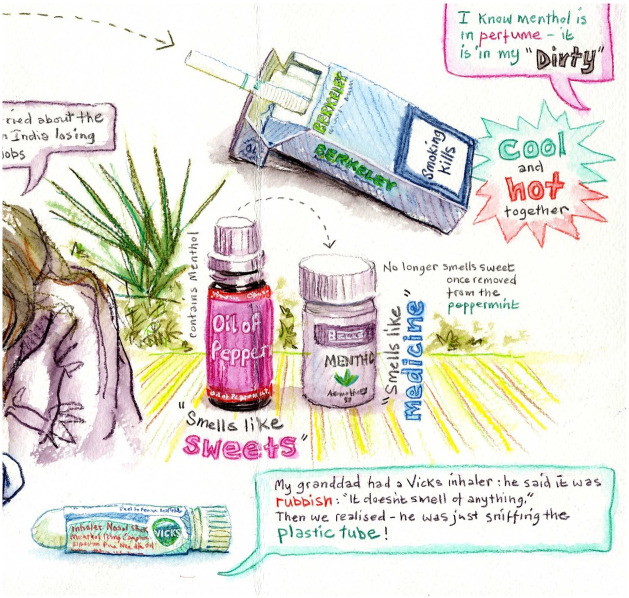
Objects used at the pop-up stalls and some of the participants’ immediate reactions.

While we are arguing that menthol is primarily connected into the moral order, and this could partly explain its surreptitious success as a tool in myriad practices, a notable way in which pleasure featured in accounts was in the immediate responses after smelling a vapour rub. Many participants exclaimed, ‘I love it!’ and a few wrinkled their noses in disapproval and displeasure. In the sketch (see Figure 5) from a pop-up stall, Lynne (the artist) has captured some of the participants’ immediate statements upon smelling the items, for example in one saying ‘it smells like sweets’ or another ‘it smells like medicine’. There were stark contrasts in people’s immediate sensory experiences of menthol products, as we might expect given the differences in taste and sense perception and so forth.

More consistent was the way in which those immediate experiences, however diverse, tended to segue into explanatory talk accounting for the way the participant had responded, which very often invoked memories of family. For example, smelling a vapour tube for delivery of menthol to the nostrils, one participant smiled and immediately told an anecdote about how her grandfather was frustrated that the menthol did not smell of anything, only to later find that he had been sniffing the plastic tube without unscrewing it.

The connections made by participants were most often to parents or grandparents or carers looking after them when they felt ill. In the following excerpt from home tour 4, Gabby, a children’s speech therapist, explains how she came to use ointments and oils and why:

I think my parents always used it, you know, it is part of what you remember, isn’t it? Being looked after at home. I cannot think of any specific time, just it was always around whenever I had a cold . . . When do I use them now? When we all have colds and we cannot breathe – usually at night, yes, at nighttime – I douse it very liberally all over the pillows, round the edges because you don’t want to lie on it and it go into your eyes – painful! . . . I put it on their chests and I have also started, and I don’t know if this is mad – you know what I am going to say! [responding to interviewer’s smile] – I have started putting it on their feet. I cannot see why that would work or how it would work but I have started doing that! . . . I think they [my children] quite like it and again they might just feel more cared for. It’s one of the only things you can do, I think, when they are really snotty and you are trying to help them get to sleep. I do think it works but even if it didn’t, it would be like, ‘Look, I am trying to do something!’ (Gabby, home tour 4)

Gabby explains how she applies ointment to her children’s torsos and feet, rubbing and massaging in a way lubricated by the waxy formulation to spread the menthol chemical out on the surface and thus to spread out of the warmth and tingling sensation. She says that she does these things to make her children aware she is caring for them, even if they do not work (objectively) to reduce symptoms directly. The laughter about using mentholated ointments on children’s feet to soothe coughs and colds is indicative of her acknowledgement of this conundrum of objectivity and the need to show her care for her children. Laughing with the interviewer’s smile shows that she knows that we, as researchers, will have encountered this practice before because it is the kind of thing that parents do in her community. Indeed, rubbing menthol on children’s feet came up frequently in our data set. While participants acknowledged this was probably not ‘really’ effective, they nonetheless used vapour rubs in this fashion in order to show – to themselves and to their children – they were taking action that might help. In this regard, it did have an effect, for here we again see the use of material things to engage the sensory as a *manifestation* of care, and how it is crucial to the relational negotiation of caring for sick children. Gabby’s talk also relates to two further processes we term *anchoring* and *conserving* moral order through use of moral things, which we now develop.

There were many such examples of people caring for their children in this fashion (it was the most dominant theme in our data). Typically, menthol is put on pillows and tissues; participants would tend to spot drops of an inhalant such as Olbas oil, which is more volatile and easy to apply to fabric. In contrast, waxy vapour rubs are used on the skin. Parents rub them on children’s body parts, often those parts with the largest open areas like the chest and back. Being on the warmer parts of the body’s core also enhances the vaporisation, meaning that more scent is given off, enhancing the sensory effects on internal bodily surfaces of the nose and throat. Indeed, the physical connection, of ‘smearing on’ and ‘rubbing in’ menthol products is a key part of manifesting care.

The temporal dimension is inextricably bound up with these uses of moral things: immediately, in terms of the physical moment in which parents rub in ointments – because it signifies care is being done (what we have termed manifesting). But also, we argue, proximally, in the hours after their use because application of waxy rubs and use of oily inhalants produces a residue of care in ways which might otherwise be difficult to achieve. Almost every parent we spoke to told us about the use of vapour rubs and similar products to look after children once they’d gone to school (rubbing under school shirts), or as they went to bed. Crucially, this is contingent upon how the menthol sensation remains in the absence of the parent or carer. For example, when the parent leaves the bedroom at night, the mentholated products still effect warming, tingly sensations on the feet or back, and a fresh, cooling sensation in the nose and throat, calming children and effecting a kind of residual parental presence.

Perhaps because of its effect on temperature sensors in the epidermis, the smell and feel of menthol can be perceived when one is bunged up with a cold while other smells and flavours are less pronounced, meaning that it is not one chemical among others, but the most important chemical for achieving this effect. The sensory aspects of menthol thus *anchor* care to particular parts of the body, to particular times within the day and to particular locations within the home. While the chemical and physiological effects of menthol on cold symptoms are debatable, and parents are often themselves unconvinced of their scientific or medical valence, the feeling that one is being cared for, and has been recently cared for, is far more important when seen from a moral practices perspective. The warming, cooling, tingling, ‘opening up’ sensations of menthol contribute across the country, night after night, for tired parents and uncomfortable children, to the alignment of a family’s evening to the extraordinary caring ideal of good parenting and of a good childhood.

**Figure 6.**
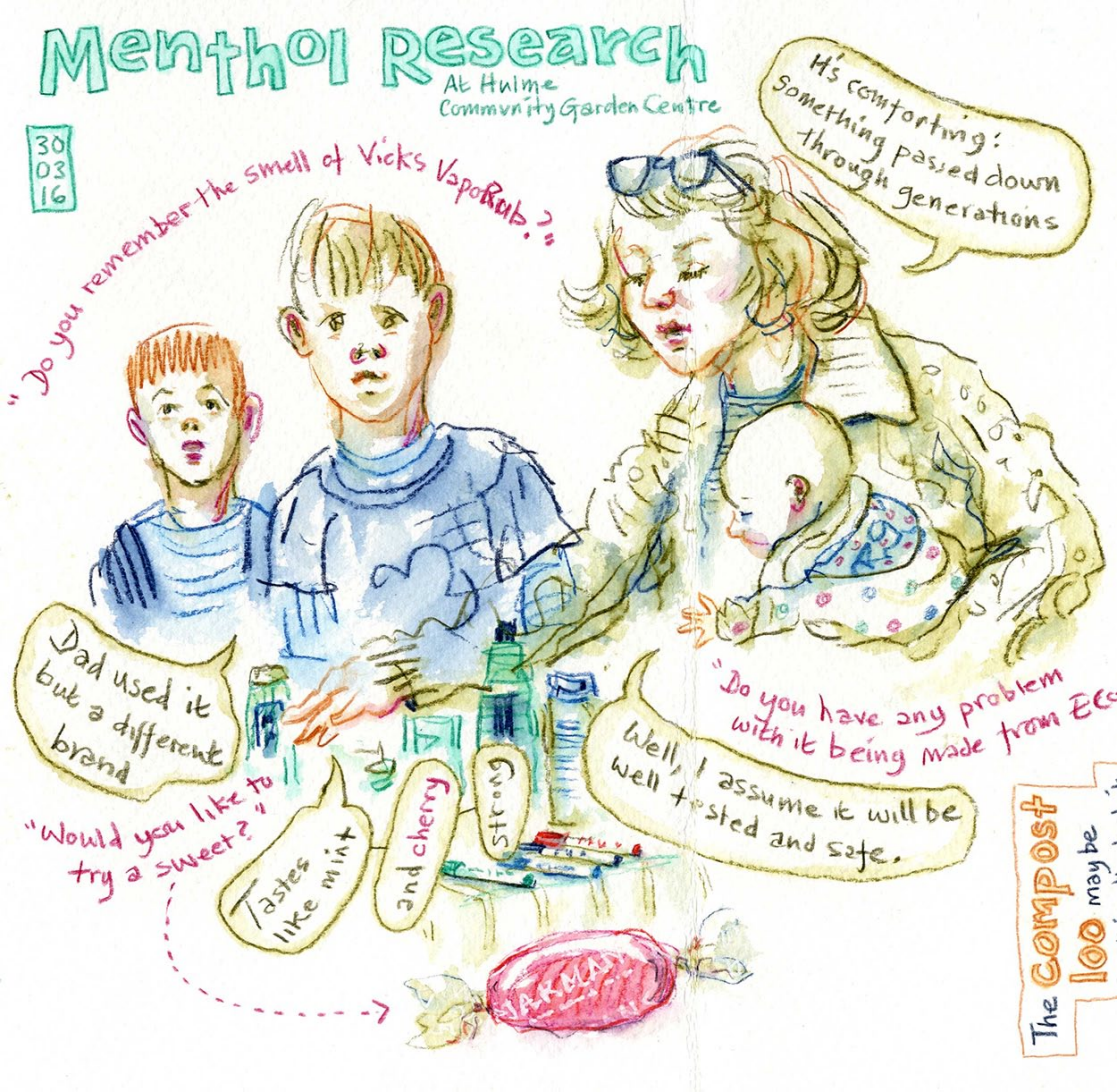
A mother and her three children engage with pop-up stall objects.

It is with respect to the idea of a good childhood and of what it means to be good parents that the concept of *conserving* moral relations emerges in our data. As a visitor to a pop-up stall wrote, ‘Vapour rub reminds me of being poorly as a child! My mum used to rub it on my chest. This is a good memory!’ (Male, 28, pop-up postcard, 18 November 2016). The memories, of being looked after when unwell, were the most frequently occurring stories told by participants in home tours, interviews and at the pop-up stalls, indicating the prevalence of this specific sensory memory in the context of menthol products’ uses. Some participants expressly made the connection of their own memory of childhood uses of menthol products to their use within tradition. For example, a mother with her three children at a pop-up stall (Figure 6) told us how vapour rub is ‘comforting; something passed down through generations’ and how she hoped her own children would go on to use it with their kids in time to come. We argue that the use of menthol products in this way *conserved* a certain idea of being cared for, across generations, by their specific sensory qualities.

**Figure 7.**
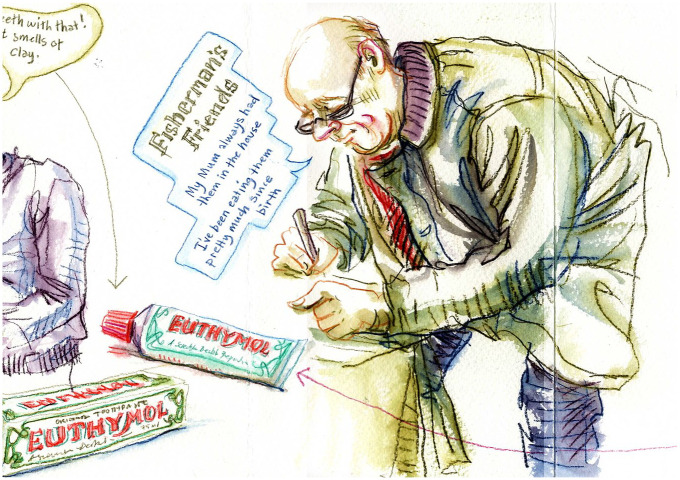
An older man describes eating menthol lozenges his whole life.

This becomes more visible by contrast, for it was rare for people to talk about remembering childhood uses of toothpaste, which no doubt they did have to be taught to use, or of parents smoking menthol cigarettes, which again must have been quite a common experience for many participants. All of these products and more were available to them at the pop-up stalls and in their own homes during our home tours. But vapour rubs were by far and away the products most frequently associated with childhood memories. There were some other examples of passing on preferences for menthol products. An older man at a pop-up stall in a garden centre (Figure 7) recalled his mother sucking on a particular brand of menthol lozenge, which he said meant he had enjoyed them too, throughout his life: ‘I’ve been eating them pretty much since birth.’

**Figure 8.**
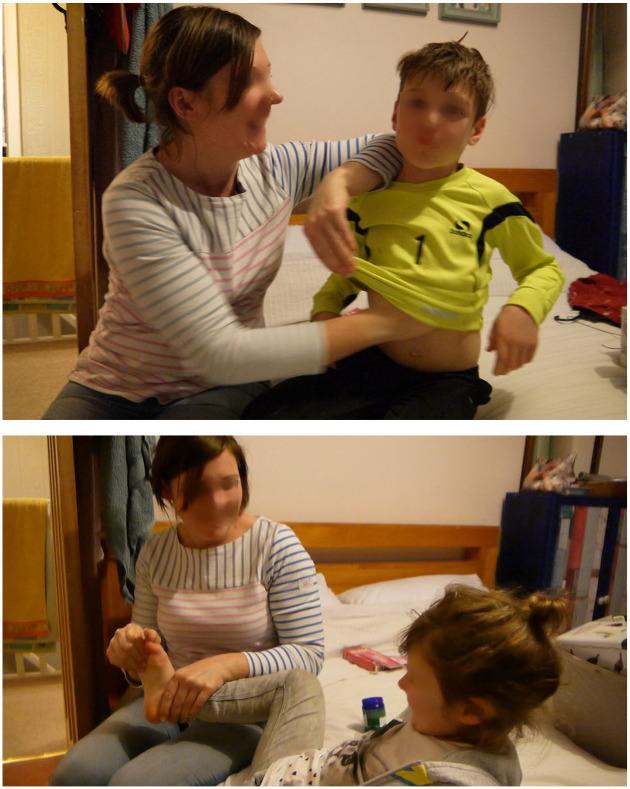
Video stills as Jen smears vapour rub onto her son’s chest and onto her daughter’s feet.

What appears to distinguish vapour rubs from other menthol products such as lozenges, in this regard, are their particular physical, relational role in the practices of care for children, because they are applied to the body by a parent or guardian in the anchoring process described above. In Figure 8, video stills show how Jen smears menthol rub onto her son’s chest and onto her daughter’s feet.

In this video, the children laugh and wriggle around, and Jen smiles and laughs with them, in an endearing caring encounter. As Jen puts the vapour rub onto her daughter, Jessica, she asks her whether it feels nice. Jessica says it is tickly and cool, and makes her feel like she is playing on the moon. There is lots of laughter at this because it is an unexpected response. Still, it speaks to the fun and safe nature of the encounter with menthol that it feels like playing, for Jessica, and perhaps the moon is invoked because it is cold. We could speculate, too, that this dream-like vision is associated with the night-time routine of putting vapour rub onto Jessica’s body before she goes to sleep, to dream of adventures in the lunar landscape.

As the children move off the bed to go and play a little, the researcher asks Jen why she uses the vapour rub like this when the children are ill. Jen explains that she just uses it because it is nice and was pleasant when she was young:

It is quite a pleasant smell although it is quite overpowering, it is quite a nice smell. I think it’s, I just associate it with feeling better as a child. So probably I just think ‘oh that’s what my children need’.

Almost every parent we encountered, in hundreds of moments in pop-ups and dozens more in substantive interviews, described a similar sense of putting vapour rub on children because they remember how it felt nice as a child. Attempts to conjure some of the same pleasant sensations and happy experiences of care one had as a child are part and parcel of parenting practices. Vapour rubs become moral things within these reproductions of childhood caring practices since they help to bring about the same experiences by virtue of their sensory qualities, physically connecting parents to their children, and remind parents of what it was like to be loved and looked after, confirming for them that they too are doing a good job of care. In other words, recollecting being cared *for* also appeared to carry the moral message that caring for children’s sensory experiences in these ways is good and desirable, and so these practices were then actively passed on to subsequent generations. In this way, conserving moral relations becomes possible in some circumstances by use of moral things’ sensory function within the temporal-moral order.

## Conclusion

Menthol products help to show how care practices are conducted within the sensory regime. We have argued that it is the sensory qualities of at least some menthol products that help to manifest care, making it visible and tangible, for participants in embodied relations. Through the use of touch, taste, smell and so forth, our participants used menthol to feel and see care happening, both for themselves and others. Our study offers data which support the development of the idea of *moral things*, especially potent resources within temporal organisation of the moral order due to their material qualities within the sensorium that produce temporary, residual and biographical moral effects. We conceptualised these effects of menthol products as manifesting, anchoring and conserving moral relations within everyday life practices.

Moral things are artefacts enrolled into routine moral work in everyday life, specifically through their material, sensory qualities within the temporal sequencing of mundane practices over time (from the immediate, to the proximal and the distant) differentiating them from other things within those practices. This opens up the potential for further exploration of how other moral things might figure within everyday practices and how these moral effects shift or are concretised by virtue of myriad possible changes in the ever-shifting sensorium. This set of empirically derived concepts offers a way to gain empirical traction and think through the ways that moral practices are achieved temporally and materially more generally.

The concepts are thus of value to researchers interested in the sensory and moral aspects of everyday life. It is likely, for instance, that other flavours and fragrances are used in comparable temporal ways. Vanilla, for example, is another mundane flavour-fragrance found in a diverse array of household and consumer goods. Not only would one find it in different forms for cooking (vanilla essence bottles, vanilla pods, vanilla seeds) but also as an ingredient in things like candles, air fresheners, confectionery and foodstuffs like ice cream or yogurt, and indeed in the perfumery industry. It is thus quite conceivable that vanilla might be used to accomplish presentations of class as a moral quality. Choices in supermarkets and in home cooking might manifest class as one chooses Madagascan vanilla pods over liquid vanilla essence; a vanilla-scented candle may leave a residual odour anchoring cultural taste in the home; and the passing down of perfume choices to children, or cultivating their preferences for certain vanilla sources over others, would represent conserving sensory moral order through generations. A harder case would be to explore whether other kinds of moral things, beyond flavours and fragrances, might also be engaged in the work of manifesting, anchoring and conserving moral order, and whether these might be found in more distinct and surprising contexts, for example in stations or schools, and within sporting, or scientific practices. We have shown that care figures prominently within the context of menthol’s usages in western culture. What work might other moral things be doing within the sensory-temporal dimensions of everyday life in the myriad contexts where smell and taste and touch become salient?

Our analysis of the data we generated with participants suggests that menthol derives its successful deployment in a host of practices because it is bound up with the moral order. This particular niche in moral practices, to use an ecological metaphor, has afforded menthol a capacity for widespread distribution, especially within forms of care. Importantly, participants made sensations of menthol accountable not primarily through somatic but through moral, relational accounts, indicating both the difficulty of articulating the bodily but also providing evidence of the way in which bodily sensations are always bound up with the sensory and social, the material and biographical. This also gestures to an important further axis in which our investigations of moral things might be developed, namely with regard to how the sensory connects the moral to the economic. Menthol’s success has been achieved within particular economic relations, and we must ask whether there are broader moral and political issues at stake in how certain kinds of sensory experience become so deeply entwined with how we manifest, anchor and conserve our most intimate of expressions of self and society. What else might be manifested, what other relations anchored, what commitments conserved as we engage in making the moral sensible?

## Supplemental Material

sj-pdf-1-soc-10.1177_0038038520959263 – Supplemental material for The Temporal Uses of Moral Things: Manifesting, Anchoring and Conserving Caring Relations within the Sensorium Click here for additional data file. Supplemental material, sj-pdf-1-soc-10.1177_0038038520959263 for The Temporal Uses of Moral Things: Manifesting, Anchoring and Conserving Caring Relations within the Sensorium by Andrew Balmer, Robert Meckin and Owen Abbott in Sociology
