# Supplementary material for: The Temporal Uses of Moral Things: Manifesting, Anchoring and Conserving Caring Relations within the Sensorium
Source: Sociology. 2020 Dec 4;55(3):619–40. doi: 10.1177/0038038520959263 (PMC8188994; doi:10.1177/0038038520959263)
Supplement: sj-pdf-1-soc-10.1177_0038038520959263 – Supplemental material for The Temporal Uses of Moral Things: Manifesting, Anchoring and Conserving Caring Relations within the Sensorium [file sj-pdf-1-soc-10.1177_0038038520959263.pdf]

Online Appendix I: Summary of data collection and participants

Pop-up stalls

Date	Location	Description	Data collected
14.05.2016	Garden centre, Greater Manchester	Community garden centre with a range of people, mostly young families and senior	Photographs, Field notes, Professional sketches, 95 participant postcards
18.11.2016	University, Greater Manchester	Science department open day for 6th form students. Attendees were students, their teachers and a range of university researchers and staff	Photographs, Field notes, Short video clips, 88 participant postcards
17.03.2017	University, Greater Manchester	University introduction day for schoolchildren. Present were hundreds of schoolchildren, their teachers and various university researchers and staff	Photographs, Field notes, 109 participant postcards
18.03.2017	Museum, Greater Manchester	Themed activity day with various stalls for activities aimed at young families	Photographs, Field notes, Professional sketches 95 participant postcards
4.04.2017	Large shopping centre, Greater Manchester	Part of university community engagement with several stores dispersed throughout centre	Photographs, Field notes, 57 participant postcards

Focus Groups

Date	Location	Description	Data collected
9.12.2015	University, Greater Manchester	Pilot of object-elicitation methods; social science researchers and support staff	Field notes
17.05.2017	Climbing centre, Sheffield	Seven participants with a range of occupations – 1 retired academic, 2 nurses and 4 people in IT and financial industries	Field notes, Professional sketches, Audio recording.

Home tours

Date	Location	Participant details	Data collected
08.11.2015	Private residence	Male, 60	Audio recording (68 min)
27.11.2015	Private residence	Female, 35	Audio recording (62 min)
27.11.2016	Private residence, Sheffield	Male, 35; female, 33; male, 7; female, 5 (family)	Audio recording (79 min)<br>Photographs<br>Video clips
27.01.2017	Private residence, Sheffield	Female, 34 (children absent)	Audio recording (88 min)<br>Photographs
27.03.2017	Private residence, Greater Manchester	Female, 33; male; 35 (couple)	Audio recording (60 min)<br>Photographs
05.04.2017	Private residence, Nottingham	Female, 35 (children present but not participants)	Audio recording (51 min)<br>Photographs,<br>Video clips
25.05.2017	Private residence, Greater Manchester	Female, 26	Audio recording (85 min)<br>Photographs
25.05.2017	Private residence, Cheshire	Male, 45; female, 10 (family)	Audio recording (50 min)<br>Photographs

Object elicitation interviews

Date	Location	Participant details	Data collected
17.09.2015	University, Greater Manchester	Female, 40	Audio recording (75 min)<br>Field notes
03.03.2017	Café, Greater Manchester	Male, 18	Audio recording (63 min)<br>Field notes
06.04.2017	University, Greater Manchester	Female, 33	Audio recording (72 min)<br>Field notes
07.04.2017	University, Greater Manchester	Female, 28	Audio recording (70 min)<br>Field notes
12.04.2017	University, Greater Manchester	Female, 34	Audio recording (88 min)<br>Field notes
27.04.2017	University, Greater	Female, 39	Audio recording (74

	Manchester		min)<br>Field notes
23.02.2017	Café, Greater Manchester	Female, 49	(Audio recording (75 min)<br>Field notes
